# Research on the Disc Sensitive Structure of a Micro Optoelectromechanical System (MOEMS) Resonator Gyroscope

**DOI:** 10.3390/mi10040264

**Published:** 2019-04-19

**Authors:** Xiang Shen, Liye Zhao, Dunzhu Xia

**Affiliations:** Key Laboratory of Micro-Inertial Instrument and Advanced Navigation Technology, Ministry of Education, School of Instrument Science and Engineering, Southeast University, Nanjing 210096, China; shenxiang91@163.com (X.S.); xiadz_1999@163.com (D.X.)

**Keywords:** micro optoelectromechanical system (MOEMS), resonator gyroscope, waveguide micro-ring resonator, modal analysis, structural simulation, frequency response, optic displacement

## Abstract

A micro optoelectromechanical system (MOEMS) resonator gyroscope based on a waveguide micro-ring resonator was proposed. This sensor was operated by measuring the shift of the transmission spectrum. Modal analysis was carried out for the disc sensitive structure of the MOEMS resonator gyroscope (MOEMS-RG). We deduced the equations between the equivalent stiffness and voltage of each tuning electrode and the modal parameters. A comprehensive investigation of the influences of the structure parameters on the sensitivity noise of the MOEMS-RG is presented in this paper. The mechanical sensitivity and transducer sensitivities of the MOEMS-RG, with varying structural parameters, are calculated based on the finite-element method. Frequency response test and the fiber optic spectrometer displacement test were implemented to verify the reliability of the model. Research results indicate that the resonant frequencies of the operating modes are tested to be 5768.407 Hz and 5771.116 Hz and the resonant wavelength change ΔX was 0.08 nm for 45° rotation angle. The resonant wavelength, which has a good linear response in working range, changes from −0.071 nm to 0.080 μm. The MOEMS-RG, with an optimized disc sensitive structure, can detect the deformation of the sensitive membrane effectively, and has a high sensitivity. This resonator shows very large meff, low $f_{0}$, and very high Q. Therefore, this resonator can provide a small ${ARW}_{B}$($0.09{}^{\circ}/\sqrt{h}$), which makes it a promising candidate for a low-cost, batch-fabricated, small size inertial-grade MOEMS gyroscope. The multi-objective optimization method could be expanded to include other objectives, constraints, or variables relevant to all kinds of gyroscopes or other microelectromechanical systems devices.

## 1. Introduction

Research on micro-resonator gyroscopes has been going on for decades. Techniques such as displacement detection, signal acquisition and analysis are widely used in vibration sensing, navigation systems, and impact detection [1,2]. Compared with conventional types, sensors based on optical effects have the advantage of high sensitivity, low weight, anti-electromagnetic interference and low manufacturing cost. Optical interference-based sensors with different structures can be designed and fabricated using the principles of interference effects, fiber bragg gratings, and waveguide coupling theory [3,4].

As a new type of high-performance gyroscope, the micro optoelectromechanical system resonator gyroscope (MOEMS-RG) consists of a series of concentric rings connected through alternating spokes to a central anchor. There are a rich set of abundant internal electrodes for driving and electrostatic trimming [5]. The micro-ring resonator based on the optical waveguide structure can be integrated on a large scale due to the MOEMS process. Excellent optical properties, such as a high-quality factor and small volume, are easy to obtain by the process of the main path. Its transmission spectrum has a large free spectral width. This width allows for extremely high resolution, making it easier to produce MOEMS sensors with relatively good performance.

It is well known that the disc sensitive structure has a great impact on the performance of the MOEMS-RG. Most of the proof mass and stiffness of the MOEMS-RG is contributed by the nested rings. The varying structural parameters (including ring number, sensitive membrane offset distance and structure height) of the disc sensitive structure are critical to the sensitivity and mechanical noise of the MOEMS-RG. In addition, the manufacturing process of a micro-resonator gyroscope invariably creates random minute mass and stiffness asymmetries that cause the natural frequencies of these modes to deviate from one another, thereby degrading sensor performance, such as mechanical sensitivity. Besides, the antinode orientations of the two modes depend on the distribution of these random minute mass and stiffness. If the antinode orientation deviates from the midline of the driving electrodes, the coupling of the two modes will be strengthened. The coupling of the two modes would farther impact bias instability. The reduction of this frequency split can be achieved by mass perturbation, such as ablating mass [6,7,8] and mass deposition [9].

The theoretical model of mass perturbation for a ring has been discussed in many papers [10,11,12,13]. In most of these studies, the intuitive functional relation between mass distribution and frequency split has been derived. And the theoretical model of mass perturbation for a ring has been successfully applied on disc sensitive structure gyroscopes, as they have a similar structure. 

A MOEMS-RG based on a waveguide micro-ring resonator is introduced in this paper. This sensor operates by measuring the shift of the transmission spectrum. Modal analysis was carried out for the disc sensitive structure of MOEMS-RG. We deduce the equations between equivalent stiffness, or voltage, of each tuning electrode and the modal parameters. The concepts of the tuning model are studied to better illustrate this theory. A comprehensive investigation of the influences of the structure parameters on the sensitivity noise of the MOEMS-RG is presented. The mechanical sensitivity and transducer sensitivity of the MOEMS-RG with varying structural parameters (including ring number, sensitive membrane offset distance and structure height) is calculated based on the finite-element method. A frequency response test and the fiber optic spectrometer displacement test were implemented to verify the reliability of the model. This study can give an explicit guideline for designing all kinds of MOEMS-RGs. The multi-objective optimization method could be expanded to include other objectives, constraints or variables relevant to all kinds of gyroscopes or other microelectromechanical systems devices. 

## 2. Working Mechanism Analysis of MOEMS-RG

**Figure 1:**
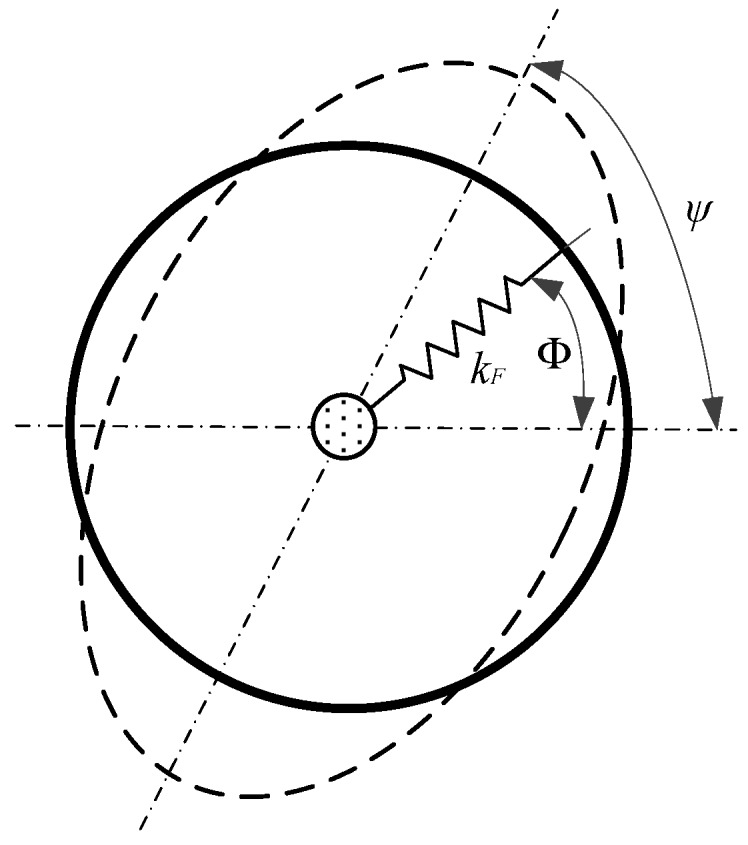
Ring with added radial springs.

The principle of modal vibration of MOEMS-RG is introduced firstly. Like all solid-state wave gyroscopes, the working mechanism of the MOEMS-RG is based on the inertia effect of the standing wave in two vibration modes of the axisymmetric resonator caused by Coriolis force. Modal refers to cases where stiffness perturbations are added on an imperfect resonator. As shown in Figure 1, radial springs are added on an ideal ring resonator, which causes the undamped natural frequency (free vibration) of the second mode to split into two different frequencies (*ω*_1_, *ω*_2_). The relationship between the stiffness perturbations and modal frequencies are reproduced below from Equations (1)–(2).

(1) $${\tan4\psi }_{j}=\frac{\sum_{j=1}^{N}(k_{F}\sin4\phi_{j})}{\sum_{j=1}^{N}(k_{F}\cos4\phi_{j})}$$

(2) $$\{\begin{array}{c} \omega_{1}^{2}=\omega_{0}^{2}(1+\alpha^{2}\sum_{j=1}^{N}(\frac{k_{F}}{{4S}_{0}}(1+\cos(4\phi_{j}-{4\psi }_{j}))))\\ \omega_{2}^{2}=\omega_{0}^{2}(1+\alpha^{2}\sum_{j=1}^{N}(\frac{k_{F}}{{4S}_{0}}(1-\cos(4\phi_{j}-{4\psi }_{j})))) \end{array}$$

where $k_{F}$ represents the stiffness of the added springs, and the corresponding angular location on the ring relative to a ring-fixed coordinate system is $\phi_{j}$, *j* = 1, 2,…, *N*. α is the amplitude ratio of the radial and tangential displacement for modes with *n* = 2 modal diameters, and $S_{0}$ is the strain energy of perfect ring in 2 nodal diameter mode; $\omega_{0}$ represents the natural frequency of the degenerate modes of the unperturbed perfect ring. We assume that the first and second order modal frequencies are $\omega_{1}$ and $\omega_{2}$, and the corresponding anti-node orientation is $\psi_{j}$; we also assume 0 ≤ $\psi_{j}$ ≤ 90°.

Thus, the frequency split can be derived as:

(3) $$\omega_{1}-\omega_{2}=\omega_{0}\frac{\alpha^{2}}{{4S}_{0}}\left[\left(\sum_{j=1}^{N}k_{F}\cos4\phi_{j}\right){\cos4\psi }_{j}+\left(\sum_{j=1}^{N}k_{F}\sin4\phi_{j}\right){\sin4\psi }_{j}\right]$$

The right-hand side of Equation (3) can be denoted as *σ_c_* and *σ_s_* respectively:

(4) $$\{\begin{array}{c} \sigma_{c}=\frac{\omega_{0}\alpha^{2}}{{4S}_{0}}\sum_{j=1}^{N}k_{F}\cos4\phi_{j}=(\omega_{1}-\omega_{2}){\cos4\psi }_{1}\\ \sigma_{s}=\frac{\omega_{0}\alpha^{2}}{{4S}_{0}}\sum_{j=1}^{N}k_{F}\sin4\phi_{j}=(\omega_{1}-\omega_{2}){\sin4\psi }_{1} \end{array}$$

Equation (4) seems to imply that the imbalance parameters of an imperfect ring are only caused by stiffness perturbations. In practice, the initial imbalance parameters are estimated by $\psi_{1}$ and ω after measuring the modal frequencies and anti-node orientations, so they include both stiffness and mass contributions to the frequency split.

**Figure 2:**
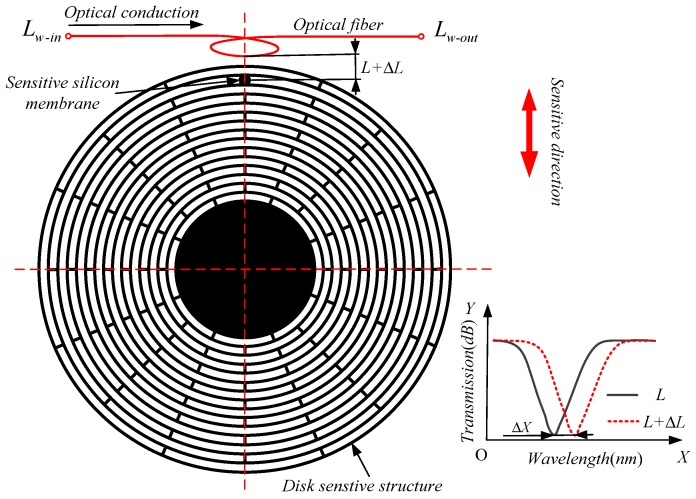
Working mechanism of the Micro Optoelectromechanical System resonator gyroscope (MOEMS-RG).

The working mechanism of the MOEMS-RG is shown in Figure 2. An evanescent wave field will be produced when light travels through the waveguide. The evanescent wave is relatively strong on the surface of the waveguide. Light is coupled into the annular microcavity in the form of an evanescent wave when the coupling parameters of annular microcavity and the optical waveguide have better matching performance. The light of the annular microcavity resonates when the frequency of the evanescent wave is equal to the eigenfrequency on the equatorial plane of the annular microcavity.

When the mode shape of the disc gyroscope changes, the distance Δ*L* of the optical fiber to the sensitive silicon membrane changes. The minimum transmission response shifts along the wavelength, and the offset value Δ*X* is positively correlated with Δ*L*. It is possible to estimate the mode shape change of the disc gyroscope and obtain its attitude change by detecting the Δ*L* value.

## 3. Modal Analysis of the MOEMS-RG

### 3.1. Establishment of the Modal Mathematical Model

**Figure 3:**
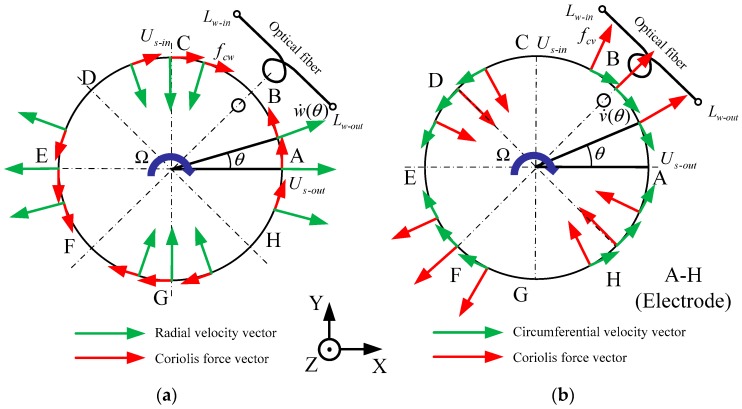
(**a**) Coriolis force vector caused by radial velocity (**b**) Coriolis force vector caused by circumferential velocity.

Modal analysis of the MOEMS-RG is carried out in this section. The working mechanism of the MOEMS-RG is based on the inertia effect of the standing wave in two vibration modes of the axisymmetric resonator caused by the Coriolis force. When a fixed frequency is applied, the MOEMS-RG vibrates in a modal direction at a certain frequency. Vibration velocity vectors and Coriolis force vectors of the active mode are shown in Figure 3.

While the resonator is in active mode, if the gyroscope is rotating about the Z axis with an angular velocity (to be measured), the Coriolis force *F_c_* in the vibrating ring, which is perpendicular to the vibration velocity vector of the active mode and the angular velocity vector. For the piezoelectric effect, the strain of piezoelectric elements produces an output signal U_s_, which is proportional to angular velocity and can be detected by the readout circuit. A-H are the positions of the electrode applied. In Figure 3, the symbol Ω represents the rotation angle of the disc and θ represents the sensitive angle of the disc; $\dot{v}$ and $\dot{w}$ represent the circumferential velocity vector and the radial velocity vector, respectively.

The dynamic radial and circumferential displacement functions of the ring in active mode are subjected to Equation (5) based on a dynamic magnification method [14,15,16,17,18,19,20].

(5) $$\{\begin{array}{c} v_{a}(\theta ,t)=v_{0}\frac{1}{\sqrt{{(1-v_{a}^{2})}^{2}+4\xi^{2}v_{a}^{2}}}\cos2\theta \sin(pt-\phi_{a})\\ w_{a}(\theta ,t)=w_{0}\frac{1}{\sqrt{{(1-v_{a}^{2})}^{2}+4\xi^{2}v_{a}^{2}}}\cos2\theta \sin(pt-\phi_{a}) \end{array}$$

in which *v_a_* = *p*/*ω*_a_ is the frequency ratio, $\phi_{a}=\arctan2\xi v_{a}/(1-v_{a}^{2})$ is the phase angle, and $1/\sqrt{{\left(1-v_{a}^{2}\right)}^{2}+4\xi^{2}v_{a}^{2}}$ is dynamic magnification coefficient. If the active frequency of the voltage applied on piezoelectric element is exactly equivalent to the natural frequency of the active mode. Then, *v_a_* = 1 and $\phi_{a}$ = *π*/2*a*. Therefore, the displacement functions of the ring in active mode can be simplified as the following form:

(6) $$\{\begin{array}{c} v_{a}(\theta ,t)=v_{0}\frac{1}{2\xi }\sin2\theta \sin(\omega_{a}t-\frac{\pi }{2})\\ w_{a}(\theta ,t)=w_{0}\frac{1}{2\xi }\cos2\theta \sin(\omega_{a}t-\frac{\pi }{2}) \end{array}.$$

According to Equation (6), the vibration velocity functions of the ring in active mode can be expressed by:

(7) $$\{\begin{array}{c} {\dot{v}}_{a}(\theta ,t)=v_{0}\frac{1}{2\xi }\omega_{a}\sin2\theta \sin\omega_{a}t={\dot{v}}_{0}\sin2\theta \sin\omega_{a}t\\ {\dot{w}}_{a}(\theta ,t)=v_{0}\frac{1}{2\xi }\omega_{a}\cos2\theta \sin\omega_{a}t={\dot{w}}_{0}\cos2\theta \sin\omega_{a}t \end{array}.$$

While the gyroscope is rotating about the Z axis in clockwise at the angular velocity, there are Coriolis forces acting on the vibrating ring, and the forces caused by the radial vibration velocity and the circumferential vibration velocity on each infinitesimal element can be formulated by the Coriolis force definition:

(8) $$\{\begin{array}{c} f_{cw}\left(\theta ,t\right)=\frac{md\theta }{\pi }{\dot{w}}_{a}\left(\theta ,t\right)\Omega =\frac{m\Omega }{\pi }{\dot{w}}_{0}\cos2\theta \sin\omega_{a}td\theta \\ f_{cv}\left(\theta ,t\right)=\frac{md\theta }{\pi }{\dot{v}}_{a}\left(\theta ,t\right)\Omega =\frac{m\Omega }{\pi }{\dot{v}}_{0}\sin2\theta \sin\omega_{a}td\theta \end{array}.$$

According to the definition of Coriolis force, the Coriolis force vectors caused by the radial vibration velocity are in a circumferential direction, and the Coriolis force vectors caused by the circumferential vibration velocity are in a radial direction. The distribution of vibration velocity vectors and Coriolis force vectors is shown in Figure 3.

The static radial displacement *w_c_*_0_ and circumferential displacement *v_c_*_0_ of the ring caused by the static Coriolis force *f_cw_* and *f_cv_* are subjected to Equation (9) based on plate and shell theory.

(9) $$\{\begin{array}{c} v_{c0}=-\left(\frac{m{\dot{w}}_{0}\Omega }{4k_{F}\pi }+\frac{m{\dot{v}}_{0}\Omega }{2k_{F}\pi }\right)\frac{R^{3}}{EI_{1}}\\ w_{c0}=-\left(\frac{m{\dot{w}}_{0}\Omega }{2k_{F}\pi }+\frac{m{\dot{v}}_{0}\Omega }{k_{F}\pi }\right)\frac{R^{3}}{EI_{1}} \end{array}$$

in which *R* is the neutral radius of the ring, *I*_1_ is the inertia moment of the ring’s entire cross-sectional area computed about the neutral axis, and *E* is Young’s modulus; *k*_F_ is the radial tensile stiffness coefficient. Therefore, the displacement functions of the ring in sense mode can be simplified as the following form:

(10) $$\{\begin{array}{c} v_{a}(\theta ,t)=v_{c0}\frac{1}{2\xi }\cos2\theta \sin(\omega_{a}t-\frac{\pi }{2})\\ w_{a}(\theta ,t)=w_{c0}\frac{1}{2\xi }\sin2\theta \sin(\omega_{a}t-\frac{\pi }{2}) \end{array}.$$

### 3.2. The Modal Simulation Analysis of MOEMS-RG

The modal of the MOEMS-RG is built by changing Young’s modulus and the density of several elements, so that the frequency split caused by both mass perturbation and stiffness perturbation is simulated, and relative parameters can be obtained from Equation (4). The frequency split and the parameters of the sample resonator are listed in Table 1.

**Figure 4:**
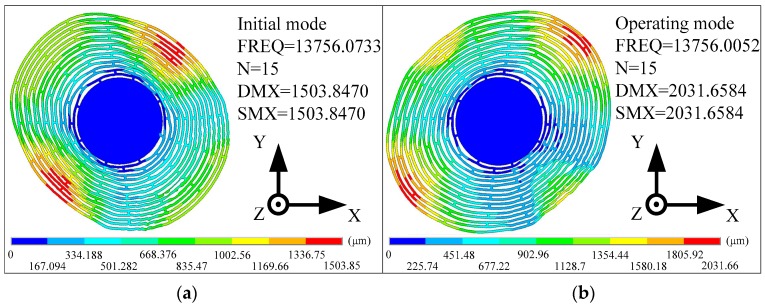
The initial modes (**a**) and the operating modes (**b**) of the sample resonator.

Mathematical simulations of the sample resonator discussed in Section 3.1 are carried out successfully and summarized in Table 1; These simulations show the frequency split achieved after setting the values of *k*_F_ by solving Equation (9). The results of modal analysis before and after tuning of the sample resonator are shown as Figure 4. Where FREQ represents the natural frequency. DMX represents the Displacement Max, and SMX represents the Solution Max. N represents the ring number. In the initial mode, the maximum positive displacement is at −X and Y deflection, and the maximum negative displacement is at X and Y deflection. Otherwise, the maximum positive displacement is at X and Y deflection, and the maximum negative displacement is at −X and Y deflection in the operating mode. The maximum displacement of the modal is higher than the initial modality. These simulations reveal that the stiffness perturbations guided by our algorithm can effectively reduce the frequency split. The residual frequency detuning can be further reduced by using the iterative method.

**Figure 5:**
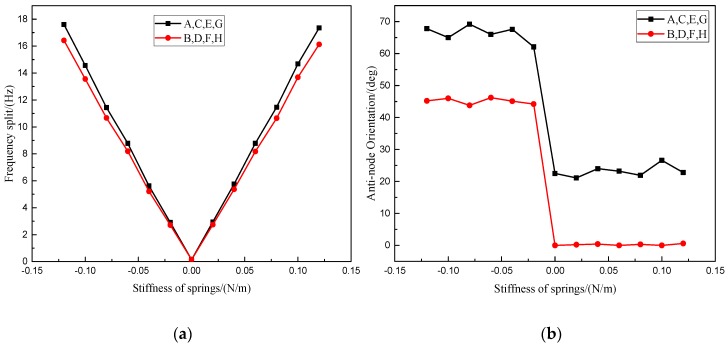

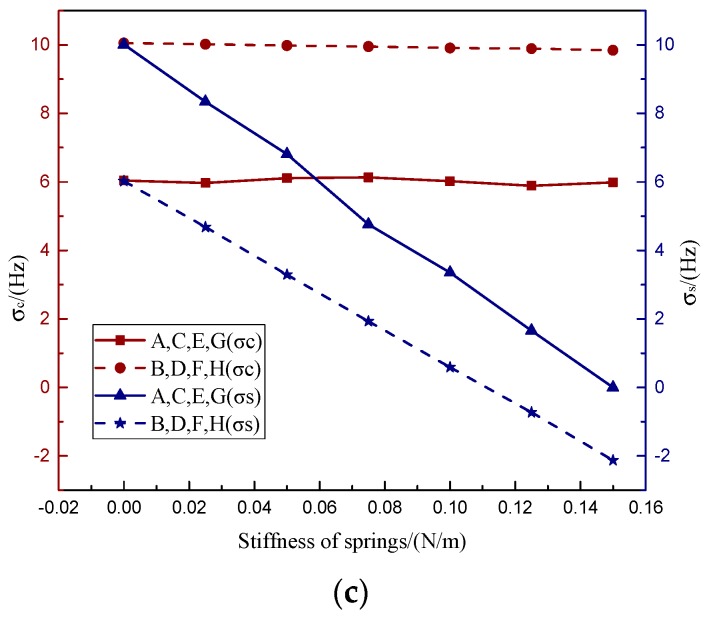
(**a**) Frequency split as function of the equivalent stiffness on corresponding electrodes (**b**) Anti-node orientations as function of the equivalent stiffness (**c**) Simulation results of reducing the imbalance parameters on MOEMS-RG.

The ideal resonator model is built through meshing symmetrically. Then, equivalent stiffness *k*_F_ is applied on a single electrode in a given step, and the equivalent stiffness of other electrodes is 0, and the frequency difference and antinode orientation are measured respectively. Finally, simulation results on electrode A and B are shown in Figure 5a,b and reveal that when *k*_F_ > 0, the corresponding frequency split increases linearly with *k*_F_, and the anti-node orientation *ψ*_1_ consistent with the centroids of corresponding electrodes; when *k*_F_ < 0, the corresponding frequency split increases linearly with the absolute value of *k*_F_, and the anti-node orientation *ψ*_1_ deviates from the centroids of the corresponding electrodes by 45°.

In addition, when *k*_F_ > 0, the slopes of these straight lines shown in Figure 5a equal to the sensitivity parameters. According to the symmetry of the resonator, electrodes on the same layer have the same sensitivity parameter, and an intuitive conclusion, which has been verified by the simulations shown in Figure 5b, is that the sensitivity parameter of the outer electrodes is greater than those with a more inboard position.

Clearly, the imbalance parameters are all positive. Thus, according to Equation (4), stiffness is applied on the equivalent springs of electrode A, C, E and G to reduce the imbalance parameter *σ_c_*. The stiffness is increased in a given step, and the imbalance parameters are calculated after each increase of the stiffness. Finally, the results are shown in Figure 5c. These results reveal that the imbalance parameter *σ_c_* decreases linearly as the stiffness increases. Further, as the stiffness increases, *σ_s_* remains unchanged. Similarly, in order to reduce *σ_s_*, stiffness is applied to electrodes B, D, F, H.

## 4. Disc Sensitive Structure Analysis of MOEMS-RG

### 4.1. Disc Sensitive Structure Design of MOEMS-RG

**Figure 6:**
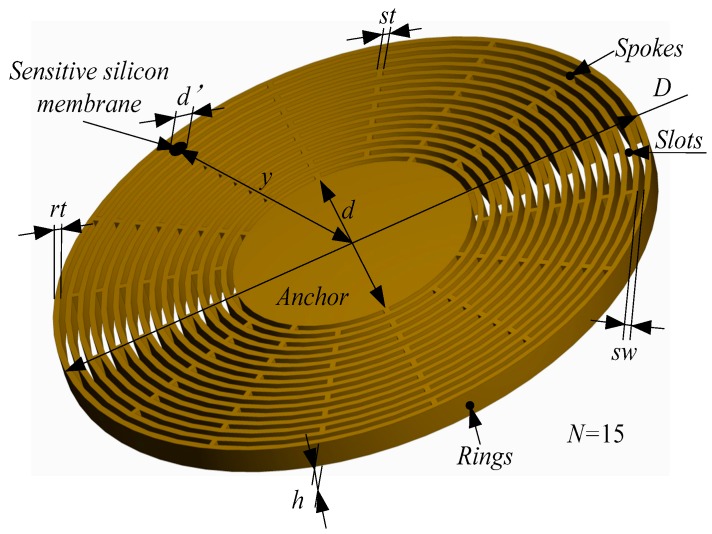
The structural schematic of the disc sensitive structure of MOEMS.

The disc sensitive structure of the MOEMS-RG is shown in Figure 6. The disc sensitive structure consists of several concentrically nested rings interconnected through 16 spokes [21]. The resonator is suspended from a single central anchor. The ring number is *N*. The outer diameter of the MOEMS-RG is *D*. The anchor diameter is *d*. The structure height is *h*. The ring thickness is *rt*. The spoke thickness is *st*. The width of the slots is *sw*. The MOEMS-RG uses the *n* = 2 wine-glass modes as the driving and sensing mode. *d*’ represents the diameter of sensitive silicon membrane and *x* is the distance from the center of the sensitive silicon membrane to anchor.

### 4.2. Mathematical Modal Analysis of the MOEMS-RG

#### 4.2.1. Mathematical Modal Analysis of a Rigid Body

**Figure 7:**
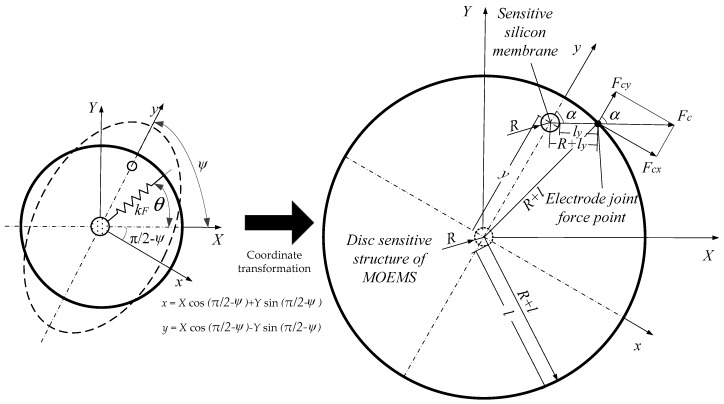
Schematic diagram of the force model of a rigid body.

In order to simplify the research, the gyroscope is analyzed as a rigid body firstly because the deformation of the disc has little effect on the study of the working range of the MOEMS disc resonators. Figure 7 represent a schematic diagram of the force model of a rigid body. The (*X*, *Y*) coordinate was converted into a (*x*, *y*) coordinate. The transformation relationship is expressed as Equation (11):

(11) $$\{\begin{array}{c} x\;=X\cos(\frac{\pi }{2}-\psi )+Y\sin(\frac{\pi }{2}-\psi )\\ y\;=X\cos(\frac{\pi }{2}-\psi )-Y\sin(\frac{\pi }{2}-\psi ) \end{array}$$

As shown in the Figure 7, *R* is the diameter of sensitive silicon membrane. l is the distance between the edge of resonator. Therefore, the radius of the resonator is *R* + *l*, Assuming that the sensitive silicon membrane is originally at the center of the gyroscope, the offset is *y* and the electrostatic force angle is *α*. *l_y_* is the distance of the sensitive silicon membrane from the edge of the large disc along the electrostatic force direction.

Clearly, the excitation voltage is composed of a DC voltage and an AC voltage. The general form of the voltage is *V*_0_ ± *V*_1_sin*ωt*. The frequency *ω* is usually much larger than the frequency of the measurement signal and the natural frequency of the mechanical structure itself. Therefore, the frequency is applied to the electrode. The force is the average value of the voltage electrostatic force and is shown as follows:

(12) $$F_{c}=\frac{{A\varepsilon \varepsilon }_{0}}{2{\left(l-y\right)}^{2}}\left(V_{0}^{2}+V_{1}^{2}/2\right)$$

The effective voltage *V* is defined as *V* $=\sqrt{\left(V_{0}^{2}+V_{1}^{2}/2\right)}$. The electrostatic force applied to the electrode is in the form of:

(13) $$F_{c}=\frac{{A\varepsilon \varepsilon }_{0}}{2{\left(l-y\right)}^{2}}V^{2}.$$

According to the Figure 7:

(14) $${\left(R+l\right)}^{2}=y^{2}+{\left(R+l_{y}\right)}^{2}-2y\left(R+l_{y}\right)\cos(\pi -\alpha )$$

(15) $$l_{y}=-R-y\cos\alpha +\sqrt{{\left(R+l\right)}^{2}-y^{2}{\left(\sin\alpha \right)}^{2}}.$$

When the gyroscope is subjected to the static acceleration signal *a*, its deformation is ignored, according to the static balance:

(16) $$\int_{-\frac{\pi }{2}}^{\frac{\pi }{2}}\frac{\varepsilon_{0}{hRV}^{2}}{2{\left(l-y\cos\alpha \right)}^{2}}d\alpha -\int_{\frac{\pi }{2}}^{\frac{3\pi }{2}}\frac{\varepsilon_{0}{hRV}^{2}\cos(\pi -\alpha )}{2{\left(l-y\cos\alpha \right)}^{2}}d\alpha +ma-ky\;=\;0$$

(17) $$\frac{\partial }{\partial y}\left[\begin{array}{c} \frac{{4\varepsilon }_{0}{hRV}^{2}\arctan\left(\frac{l+y}{\sqrt{l^{2}-y^{2}}}\right)}{\sqrt{l^{2}-y^{2}}}-\\ \frac{{2\varepsilon }_{0}{hRV}^{2}\left(2\arctan\left(\frac{l+y}{\sqrt{l^{2}-y^{2}}}\right)-\mathrm{csg}n\left(\frac{l+y}{\sqrt{l^{2}-y^{2}}}\right)\pi \right)}{\sqrt{l^{2}-y^{2}}} \end{array}\right]+ma-ky\;=\;0$$

in which, sg*n*(*y*) = [1, (if *y* > 0); 0, (if *y* = 0); −1, (if *y* < 0)]. *y* is the offset distance of the silicon sensitive membrane, *ε*_0_ is the vacuum dielectric constant and *h* is the thickness of the gyroscope chassis. *R* is the radius of the silicon sensitive membrane; *α* is the angle of the electrostatic force and *l_y_* is the distance of the sensitive silicon membrane from the edge of the large disc along the electrostatic force direction.

Let $p\;=\;\frac{{\pi \varepsilon }_{0}{hRV}^{2}}{{2kl}^{3}}$, $q\;=\;\frac{ma}{kl}$, $r\;=\;\frac{y}{l}$, the Equation (17) can be simplified as:

(18) $$\frac{2pr}{(1-r^{2})}+q-r=0$$

and when a voltage is applied, defining the function:

(19) $$q\;(r,p)=r\left(1-\frac{2p}{{(1-r^{2})}^{\frac{3}{2}}}\right)$$

**Figure 8:**
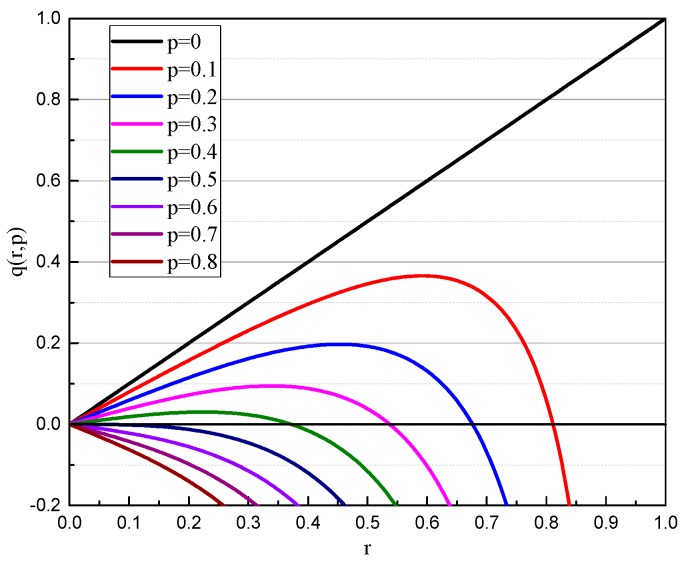
*q*(*r*,*p*) curves for undeformed gyroscope.

Equation (19) can be described in Figure 8. As can be seen from Figure 8, the curves have a maximum value *q*_max_ for a specific value *p*. When *q* ≥ *q*_max_, *r* has no real solution, which means that the gyroscope is attracted under the combined action of electrostatic force and inertial force. If *q* ≥ 0.5, there is no stable solution, which means that the gyroscope is unstable. In a certain situation, when *p* = 0.1 and *q* = 0.2, there are two intersections in the range of [0,1], in which, *r*_1_ = 0.2569, *r*_2_ = 0.7623, respectively.

Let ∂q/∂r = 0 to obtain Equation (20):

(20) $${\left(1-y^{2}\right)}^{5}={4p}^{2}{\left(1+2y^{2}\right)}^{2}$$

and when combined with Equation (11), Equation (21) can be obtained:

(21) $${\left(1-{(X\cos(\frac{\pi }{2}-\psi )-Y\sin(\frac{\pi }{2}-\psi ))}^{2}\right)}^{5}={4p}^{2}{\left(1+2{\left(X\cos(\frac{\pi }{2}-\psi )-Y\sin(\frac{\pi }{2}-\psi )\right)}^{2}\right)}^{2}.$$

#### 4.2.2. Mathematical Modal Analysis of an Elastomer Body

Based on the research in Section 4.2.1, the deformation of the gyroscope was discussed in this section. According to Hooke’s law, the solid tensile strain *ε_n_* is linear with the cross-sectional stress *σ_n_* within the proportional limit within the elastic range, that is, *σ_n_* = *Yε_n_*.

**Figure 9:**
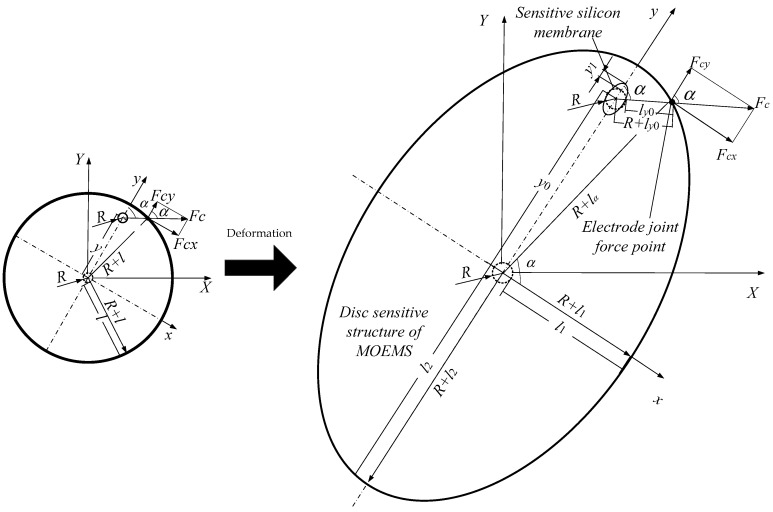
Schematic diagram of the force model of an elastomer body.

Figure 9 shows the schematic diagram of the force model of an elastomer body. As shown in Figure 9, the disc sensitive structure gyroscope is deformed by resonance. In order to simplify the calculation, it is approximately elliptical. *h* is its chassis thickness, where *y*_1_ is the shape variable of the sensitive silicon membrane, and *y*_0_ is the distance between the deformed chassis and the sensitive silicon membrane of the disc. The shape variable and the lower shape variable of the sensitive silicon membrane are simplified for the convenience of calculation. According to the same method as described above, the distance between the chassis and the sensitive silicon membrane during the offset can be found.

Also, (*R* + *l*_1_) is the ellipse short axis length, (*R* + *l*_2_) is the ellipse long axis length, and (*R* + *l_α_*) is the radius length of the angle α with the short axis; this can yield:

(22) $$l_{a}\;=\;\left(l_{1}-l_{2}\right)\sin\alpha +l_{1}.$$

It can be known from the balance of forces that:

(23) $$\int_{-\frac{\pi }{2}}^{\frac{\pi }{2}}\frac{\varepsilon_{0}{hRV}^{2}\cos\alpha }{2{\left(l_{y_{0}}-y_{0}\cos\alpha -y_{1}\cos\alpha \right)}^{2}}d\alpha -\int_{\frac{\pi }{2}}^{\frac{3\pi }{2}}\frac{\varepsilon_{0}{hRV}^{2}\cos\alpha }{2{\left(l_{y_{0}}-y_{0}\cos\alpha -y_{1}\cos\alpha \right)}^{2}}d\alpha +ma-{ky}_{0}\;=\;0$$

where *y*_0_ is the offset distance of the silicon sensitive membrane; *y*_1_ is the shape variable of the sensitive silicon membrane; *ε*_0_ is the vacuum dielectric constant; *h* is the thickness of the gyroscope chassis; *R* is the radius of the silicon sensitive membrane before deformation; *α* is the angle of the electrostatic force and l*_y_*_0_ is the distance of the sensitive silicon membrane from the edge of the large disc along the electrostatic force direction.

Simplified for Equation (23):

(24) $$\frac{\partial }{\partial x}\left[\frac{\varepsilon_{0}{hRV}^{2}}{2}-\frac{2\pi }{\sqrt{l_{y0}^{2}-{{(y}_{0}{\cos\alpha +y}_{1}\cos\alpha )}^{2}}}\right]+ma-{ky}_{0}\;=\;0.$$

Let $p=\frac{{\pi \varepsilon }_{0}{hRV}^{2}}{{2kl}_{y_{0}}^{3}}$, $q=\frac{ma}{{kl}_{y_{0}}}$, $r=\frac{y_{0}}{l}$, $s=\frac{y_{1}}{l}$, and simplified for Equation (24):

(25) $$\frac{2p(r+s)}{{(1-{\left(r+s\right)}^{2})}^{\frac{3}{2}}}+q-r\;=\;0.$$

Let *s* = 0.01, that is, *y*_1_ = 0.01*l*, obtain Equation (26):

(26) $$q\;(r,p)=r-\frac{2p(r+0.01)}{{(1-{\left(r+0.01\right)}^{2})}^{\frac{3}{2}}}$$

**Figure 10:**
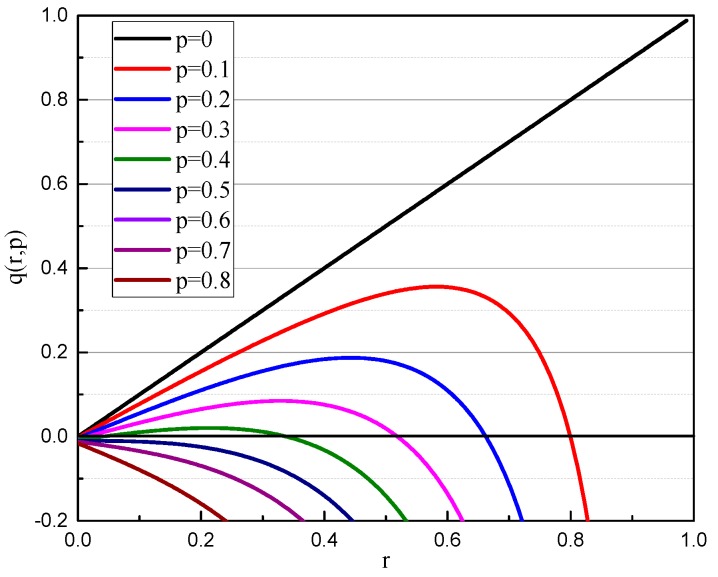
*q*(*r*,*p*) curves for the deformed gyroscope.

The *q*(*r*,*p*) curves for the deformed gyroscope are shown in Figure 10. Same as when it is not deformed, the curves have a maximum value *q*_max_ for a specific value *p*. When *q* ≥ *q*_max_, *r* has no real solution, which means that the gyroscope is attracted under the combined action of electrostatic force and inertial force. If *q* ≥ 0.5, there is no stable solution, which means that the gyroscope is unstable.

Let ∂q/∂r = 0, obtain Equation (27):

(27) $$1-\frac{2p}{{\left(1-(y+0.01)\right)}^{\frac{3}{2}}}+\frac{3p(y+0.01)(-2y-0.02)}{{\left(1-{\left(y+0.01\right)}^{2}\right)}^{\frac{5}{2}}}\;=\;0.$$

Combined with Equation (11), the following equation can be obtained:

(28) $$\begin{array}{l} 1-\frac{2p}{{(1-((X\cos(\frac{\pi }{2}-\psi )-Y\sin(\frac{\pi }{2}-\psi ))+0.01))}^{\frac{3}{2}}}+\\ \frac{3p((X\cos(\frac{\pi }{2}-\psi )-Y\sin(\frac{\pi }{2}-\psi ))+0.01)(-2(X\cos(\frac{\pi }{2}-\psi )-Y\sin(\frac{\pi }{2}-\psi ))-0.02)}{{\left(1-{\left(y+0.01\right)}^{2}\right)}^{\frac{5}{2}}}\;=\;0 \end{array}$$

**Figure 11:**
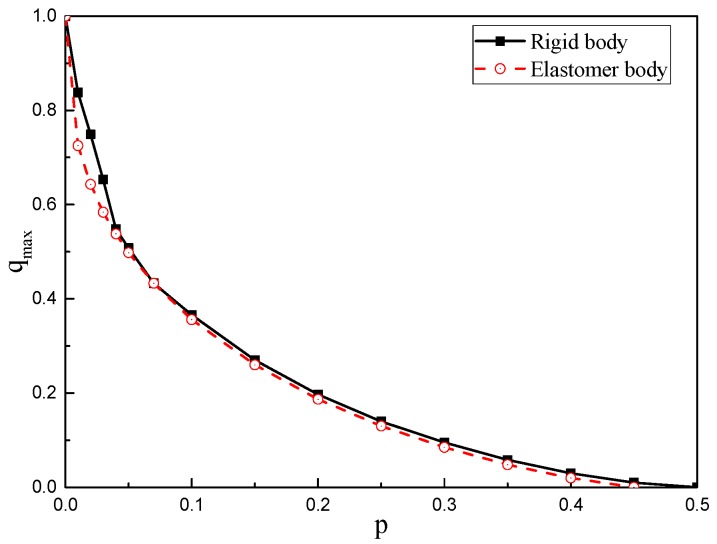
The relationship curves between *q*_max_ and *p* for rigid and elastomer bodies.

The relationship curves between *q*_max_ and *p* are shown in Figure 11. The solid line part is the relationship between *q*_max_ and *p* when neglecting deformation, and the dotted line part is the relationship between *q*_max_ and *p* when considering deformation. It can be seen from Figure 11 that when the deformation is considered, the relationship curve is located below the rigid body. The maximum value of *p* is 0.45 when the deformation spacing is 0.01, which is 0.5 when the deformation is ignored. Therefore, the deformation of the gyroscope changes its work reliability range.

## 5. Structural Simulation Analysis of the MOEMS-RG

### 5.1. Mechanical Sensitivity and Transducer Sensitivity Model of MOEMS-RG

The mechanical sensitivity *S*_mech_ is defined as the displacement amplitude of the sensing mode under each unite input angular rate, which can be expressed by [22,23]:

(29) $$S_{\mathrm{mech}}=\frac{y_{1}}{\Omega }=\frac{{4A}_{g}{Qy}_{0}}{\omega_{0}}$$

where $y_{1}$ is the shape variable of the sensitive silicon membrane, and $y_{0}$ is the distance between the deformed chassis and the sensitive silicon membrane of the disc. Ω is the input angular rate; Q is the quality factor; $\omega_{0}$ is the angular resonant frequency of the *n* = 2 working mode; $A_{g}$ is the angular gain. Generally, the drive-loop circuit will ensure that the driving amplitude stays constant. The driving amplitude is fixed when comparing the mechanical sensitivities of different MOEMS-RGs. In this study, the driving amplitude is assumed to be a constant 1 μm.

The variation of the electrode capacitor caused by the mechanical displacement of the resonator can be expressed by:

(30) $$\Delta C=\frac{A\varepsilon \varepsilon_{0}\bar{y}}{l^{2}}$$

where A and l are the capacitive area and initial gap of the sensing electrode, respectively. $\varepsilon_{0}$ is the permittivity of vacuum, and ε is the relative permittivity. $\bar{y}$ is the average displacement of the resonator region facing the electrode, which can be expressed by $\bar{y}$ = *ky*_1_. The scale factor *k* is the ratio of the average displacement and the displacement amplitude of the electrode.

The transducer sensitivity $S_{\mathrm{tran}}$ is defined as the variation of the electrode capacitor of the sensing mode under each unite input angular rate, which can be expressed by:

(31) $$S_{\mathrm{tran}}=\frac{\Delta C}{\Omega }=\frac{S_{\mathrm{mech}}{\varepsilon \varepsilon }_{0}}{l^{2}}\sum_{i}k_{i}A_{i}=\frac{{4\varepsilon \varepsilon }_{0}A_{g}{Qy}_{0}}{l^{2}\omega_{0}}\sum_{i}k_{i}A_{i}$$

where *i* is the electrode number and *k_i_* is the scale factor of each electrode, which depends on the mode shape of the resonator. For the MOEMS-RG, *k_i_* is always smaller than 1, and the outer electrode has a larger *k_i_*. $\sum_{i}k_{i}A_{i}$ can be called the effective capacitive area. In this study, the capacitive gap is assumed to be a constant 15 μm.

### 5.2. Varying Structural Parameters Simulation Analysis of the MOEMS-RG

As we know that most of the proof mass and stiffness of the MOEMS-RG is contributed by nested rings. Therefore, the number of the nested rings can greatly affect the performance of the MOEMS-RG [24]. When the ring number decreases to one, the MOEMS-RG would degenerate into the traditional resonator gyroscope. In this part, the effects of the varying structural parameters (including ring number, sensitive membrane offset distance and structure height) on the sensitivity and mechanical noise of the MOEMS-RG are discussed based on the finite-element method.

#### 5.2.1. The Ring Number

**Figure 12:**
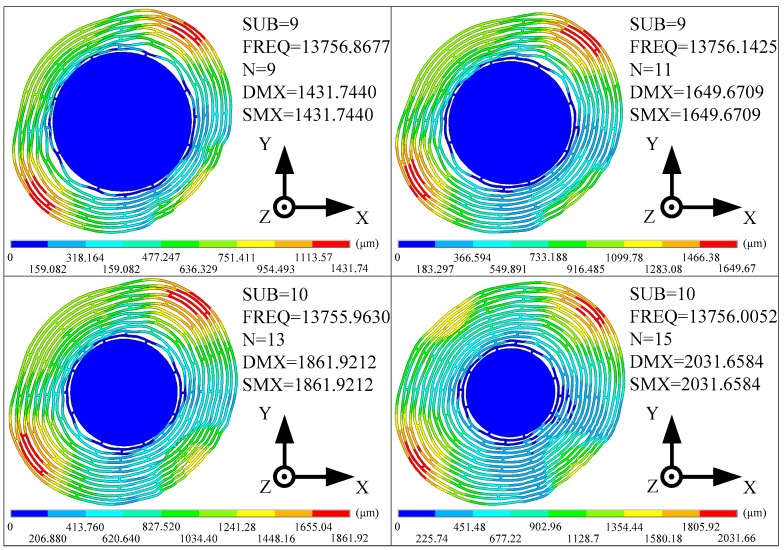
The operating modes of the sample resonator with different ring numbers.

**Figure 13:**
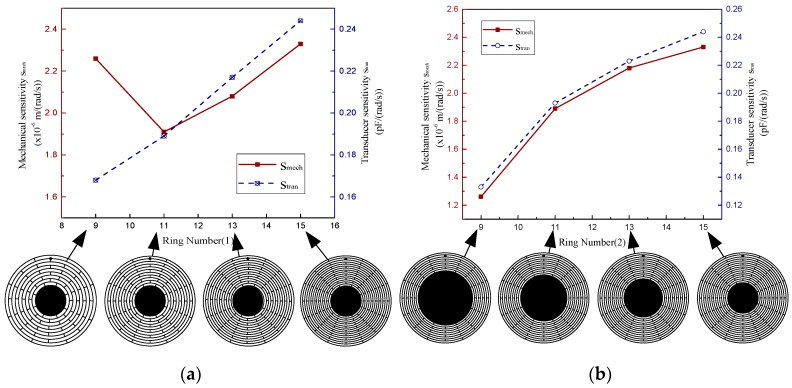
(**a**) Case 1 (**b**) Case 2.

In this part, the effects of the ring number on the sensitivity and mechanical noise of the MOEMS-RG are discussed. When the ring number is changed from 9 to 15, *S*_mech_ and *S*_tran_ of the MOEMS-RG are calculated. *S*_mech_ is calculated based on the typical electrodes sets shown in Equation (29). *S*_tran_ is calculated based on the typical electrodes sets shown in Equation (30). Two cases were discussed in this section. Firstly, adjust the ring number by changing the ring spacing *sw* with the anchor size fixed (Case 1). Secondly, adjust the ring number by changing the diameter *d* of the anchor with the ring spacing *sw* fixed (Case 2). The other structural parameters are fixed in Table 2. The simulated results are presented in Figure 12 and Figure 13. Where SUB represents the sub-iteration step. FREQ represents the natural frequency. DMX represents the Displacement Max, and SMX represents the Solution Max. N represents the ring number.

It can be seen from Figure 12 that as the ring number increases, the operating mode direction is basically the same, but the value of maximum vibration mode displacement increases significantly, and the silicon sensitive membrane becomes more sensitive.

The mechanical sensitivities and the transducer sensitivities of the MOEMS-RGs with different ring numbers are provided in Figure 13. In case one (as shown in Figure 13a), the MOEMS-RG with 11 rings has the poorest *S*_mech_. Because thermo-elastic dissipation is relatively low and natural frequency is relatively high in this region. However, *S*_tran_ of the MOEMS-RG improves with the increase of the ring number. This is due to the improvement of the effective capacitive area. In case two (as shown in Figure 13b), if the diameter *d* of the anchor decreases, both *S*_mech_ and *S*_tran_ of the MOEMS-RG increase when the ring number increases. It can be concluded that a smaller anchor size ratio should be chosen. But when the anchor size ratio is less than 0.4, other problems involving anchor loss, structural strength, and so on would emerge when the anchor size ratio is too small.

#### 5.2.2. The Offset Distance of the Sensitive Membrane

**Figure 14:**
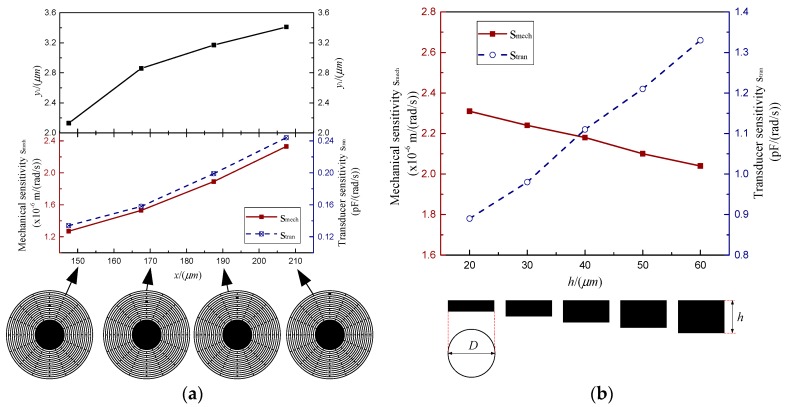
(**a**) The *y*_1_, mechanical sensitivities and the transducer sensitivities with different ring numbers (**b**) The mechanical sensitivities and the transducer sensitivities with the height of the MOEMS-RG.

The offset distance of the sensitive membrane is one of the primary factors which must be decided at the MOEMS-RG designing. It is of great importance to study how the offset distance of the sensitive membrane affects the sensitivity and mechanical noise of the MOEMS-RG. *S*_mech_ and *S*_tran_ of the MOEMS-RG with the offset distance are changed from 147.5 μm to 207.5μm are calculated. *S*_mech_ is calculated based on the typical electrodes sets shown in Equation (29). *S*_tran_ is calculated based on the typical electrodes sets shown in Equation (31). In this case, the resonator height is 40 μm, the thicknesses of the rings and spokes are 5 μm. The other structural parameters, except the slot width, are fixed in Table 2. The results are demonstrated in Figure 14a.

As is shown in Figure 14a, the shape variable of the sensitive silicon membrane *y*_1_ increased with the offset distance of the sensitive membrane *x* increasing. *S*_mech_ and *S*_tran_ of the MOEMS-RG increased simultaneously. The results show that the larger the offset distance of the sensitive membrane, the more sensitive the MOEMS-RG is.

#### 5.2.3. The height of the MOEMS-RG

The height of the MOEMS-RG is decided based on concerns such as the capacitive area and fabrication capability [25]. This part mainly studies the effects of the structure height *h* on the sensitivity and the mechanical noise of the MOEMS-RG. *S*_mech_ and *S*_tran_ of the MOEMS-RG with heights ranging from 20 μm to 60 μm are calculated. Meanwhile, the ring number is 15, The other structural parameters except the slot width are fixed in Table 2. The simulated results are summarized in Figure 14b. It can be seen from Figure 14b that *S*_mech_ decreases, whereas *S*_tran_ increases when the resonator height *h* increases. This increase is due to the enhancement of the capacitive area provided by the increase of the height.

## 6. Experiment Test of the MOEMS-RG

**Figure 15:**
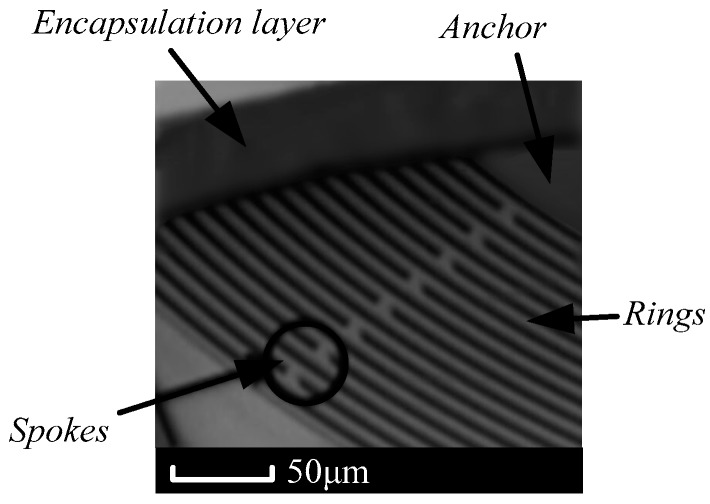
SEM image of epi-sealed MOEMS-RG.

The MOEMS-RG with optimized disc sensitive structure was processed by microelectromechanical systems (MEMS) processing methods according to Section 4.2 and Section 5. The disc sensitive structure has rings to measure the Coriolis-based rotation rate signal. An SEM image of the device is shown in Figure 15, and key design parameters are summarized in Table 2. In this work, we demonstrate that it is possible to reduce the frequency splits in silicon through slight modifications in the geometric design of the disc sensitive structures.

The epi-seal process starts with a 40 μm-thick (100) SOI (Silicon-On-Insulator) wafer with a 2 μm-thick buried oxide layer. The devices were patterned and etched using DRIE (Deep Reactive Ion Etching). Then, the trenches are filled with a 2 μm-thick sacrificial LPCVD (Low Pressure Chemical Vapor Deposition) oxide layer, and contact holes are subsequently etched into this oxide. These contact holes provide electrical access to the device structure and electrodes, creating a mechanical anchor. A first encapsulation layer (6 μm) was epitaxially grown on top of the sacrificial oxide, and vent holes were etched. Vapor-phase HF was then used to etch the oxide and release the device structure. A thick (20 μm) second encapsulation layer is deposited epitaxially to seal the vent holes and create the hermetic cavity. An aluminum for electrical contact was patterned and deposited. Final annealing was performed in a low temperature (400 °C) nitrogen environment to diffuse the residual hydrogen gas out from the cavity, providing a low pressure, oxide-free environment that is less than 10 Pa, which yields high Q devices.

**Figure 16:**
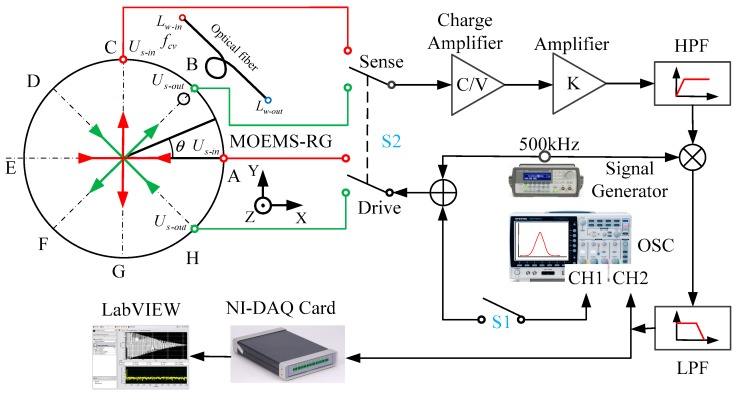
Block diagram of the experimental.

A frequency response test was implemented in a vacuum chamber at 25 °C. A block diagram of the experimental set up is shown in Figure 16. During the frequency response test, a sweeping frequency AC signal produced by the oscillator subsystem (OSC) of the frequency response analyzer is modulated into a 500 kHz carrier and used to drive the resonator. The capacitor variation of pickoff electrodes is converted into an AC output signal by a charge amplifier. The output signal is amplified and high-pass filtered (HPF) to reduce the low-frequency noise (LPF) [26]. The output signal is then synchronously demodulated by a multiplier and a low-pass filter. The driving signal and the output signal are input to the CH1 and CH2 ports of the FRA (Fiber Raman Amplifier), respectively. A double-pole double-throw switch S2 is used to change the testing axis. Signal is gathered by NI-DAQ (National Instruments Data Acquisition) card.

**Figure 17:**
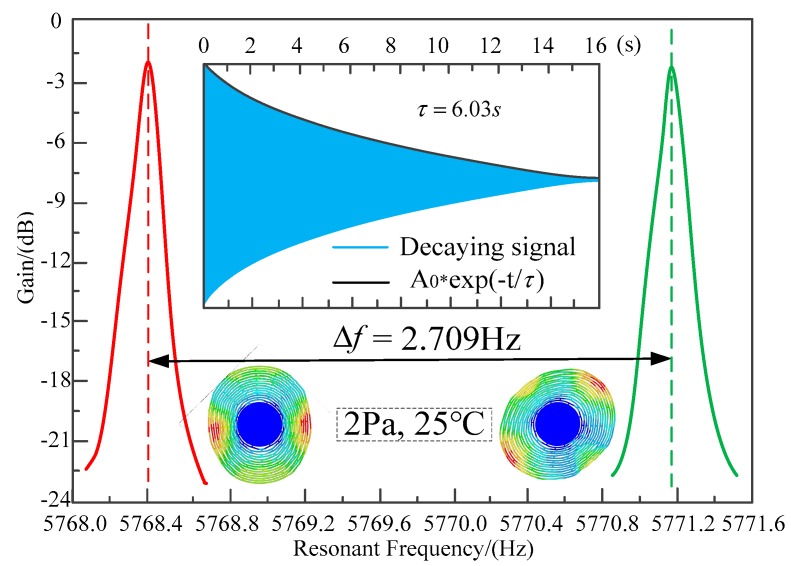
The frequency response test results.

**Figure 18:**
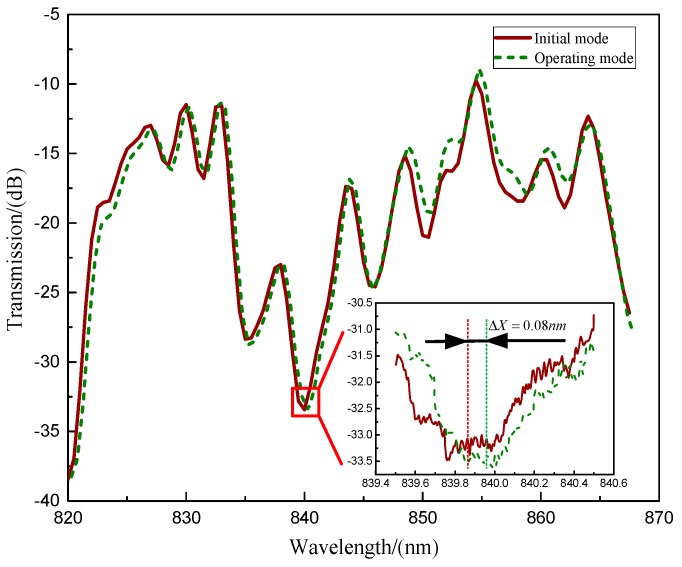
The resonant wavelength change for the operating mode.

The frequency response test results are shown in Figure 17. The resonant frequencies of the operating modes are tested to be 5768.407 Hz and 5771.116 Hz. This 2.709 Hz frequency split is caused by the nonuniformity of the material and asymmetry in the fabrication. The fiber optic spectrometer displacement test was also performed. When the MOEMS-RG vibrates in the operating mode, the silicon sensitive membrane is free to move and deform in the direction of the vibration mode. The distance Δ*L* of the optical fiber to the sensitive silicon membrane changes. Light from the wide spectrum super-luminescent diode (SLD) laser is fed into the displacement meter through a polarization controller (PC). The output from the wavelength shift was monitored by an optical fiber spectrometer (OSA, Avaspec-2048 with a resolution of 0.04 nm) to measure the displacement Δ*L*, as shown in Figure 18. The resonant wavelength change Δ*X* for the applied displacement Δ*L* was 0.08 nm for a 45° rotation angle. 

**Figure 19:**
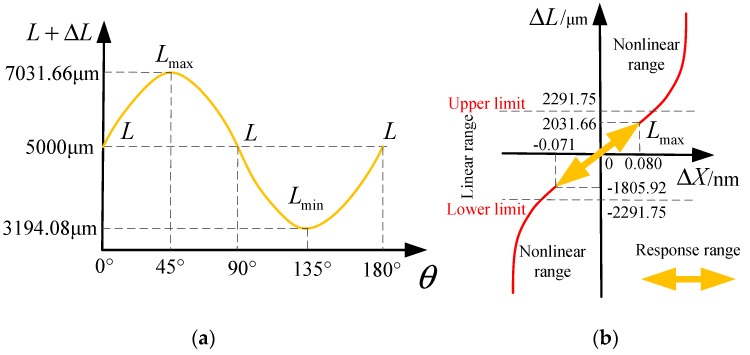
Dynamic response of the MOEMS-RG (**a**) The relationship curve of displacement of the sensitive silicon membrane ΔL and rotation angle of the resonator gyroscope. (**b**) The relationship curve of the resonant wavelength change ΔX and input signal ΔL.

Dynamic response of the MOEMS-RG was also tested in this paper. The results are shown in Figure 19. From Figure 19, we can know that the working range of the MOEMS-RG covers 0° to 180°. The response range of ΔL is from −1805.92 μm to 2031.66 μm, which is within the linear range [−2291.75, 2291.75] μm. The resonant wavelength changes from −0.071 nm to 0.080 μm. The MOEMS-RG has a good linear response in the working range.

The effective mass $m_{\mathrm{eff}}$ and Coriolis coupled mass γ of the stiffness-mass decoupled disk resonator’s n = 2 mode can be calculated using finite element analysis [27]. The angular gain $A_{g}$ of the n = 2 mode can also be calculated based on $A_{g}=\gamma /2m_{\mathrm{eff}}$. $m_{\mathrm{eff}}$, γ, and $A_{g}$ are calculated to be 2.71 mg, 2.16 mg, and 0.4, respectively. The proposed resonator is very easy to actuate. The driving amplitude of 2.5 μm can be actuated by applying only a 1.5V DC voltage and a 1.5V AC voltage. The ${ARW}_{B}$ of the resonator is calculated to be $0.09{}^{\circ}/\sqrt{h}$ based on:

(32) $${ARW}_{B}\;=\sqrt{\frac{m_{\mathrm{eff}}k_{B}T_{0}}{{2\pi f}_{0}x_{0}^{2}\gamma^{2}Q}}\left(\frac{180}{\pi }\times 60{}^{\circ}\right)/\sqrt{h}$$

where $x_{0}$ is the driving amplitude (2.5 μm), $k_{B}$ is the Boltzmann’s constant (0.0259 eV) and $T_{0}$ is the absolute temperature of the environment (298.15K). Related parameters are listed in Table 3.

This design concept exploits the size effect. A stiffness-mass decoupled disk resonator for gyroscopic application is demonstrated. This resonator shows very large meff, low $f_{0}$, and very high Q. Therefore, this design can provide a small ${ARW}_{B}$($0.09{}^{\circ}/\sqrt{h}$), which makes it a promising candidate for a batch-fabricated, low cost, small size inertial-grade MOEMS gyroscope.

**Figure 20:**
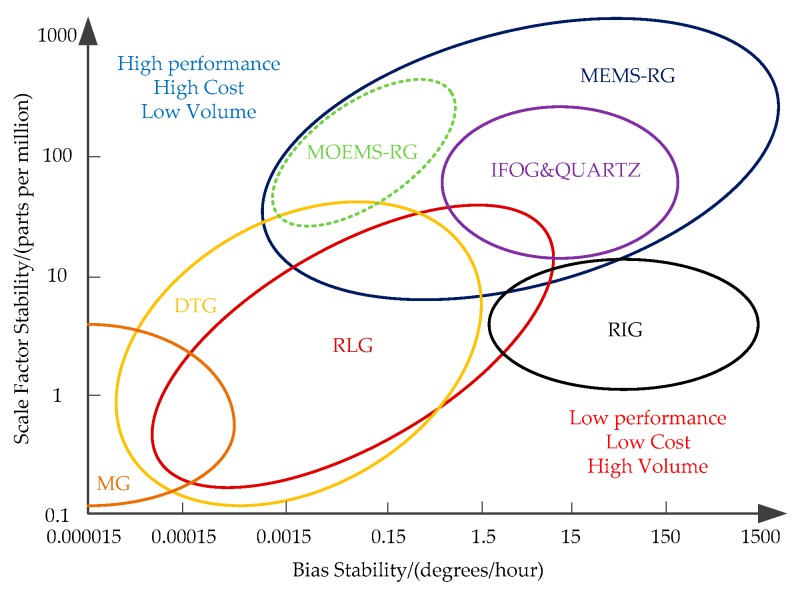
Performance comparison of various types of resonator gyroscope. Mechanical Gyroscopes (MG), Ring Laser Gyroscopes (RLG), Interferometric Fiber-Optic Gyroscopes (IFOG), Quartz, Dynamically Tuned Gyroscopes (DTG), Rate and Integrating Gyroscopes (RIG), Micro Electromechanical System Resonator Gyroscope (MEMS-RG) and MOEMS-RG.

Figure 20 shows the performance comparison of various types of resonator gyroscopes. Where scale factor stability is expressed in parts per million, as a function of the bias stability for Mechanical Gyroscopes (MG), Ring Laser Gyroscopes (RLG), Interferometric Fiber-Optic Gyroscopes (IFOG), Quartz, Dynamically Tuned Gyroscopes (DTG), Rate and Integrating Gyroscopes (RIG), Micro Electromechanical System Resonator Gyroscope (MEMS-RG) and MOEMS-RG. It can be seen from Figure 20 that MOEMS-RGs have a superior bias stability and scale factor stability. The MOEMS-RG is expected to achieve the requirements of high performance and low volume.

## 7. Conclusions

A MOEMS-RG based on a waveguide micro-ring resonator was proposed. This sensor operates by measuring the shift of the transmission spectrum. Modal analysis was carried out for the disc sensitive structure of a MOEMS-RG. We deduce the equations between the equivalent stiffness or voltage of each tuning electrode and the modal parameters. And the concepts of the tuning model are introduced to better illustrate this theory. A comprehensive investigation of the influences of the structure parameters on the sensitivity noise of the MOEMS-RG was present in this paper. The mechanical sensitivity and transducer sensitivity of MOEMS-RG with varying structural parameters (including ring number, sensitive membrane offset distance and structure height) are calculated based on the finite-element method. A frequency response test and the fiber optic spectrometer displacement test were implemented to verify the reliability of the model. Research results indicate that the resonant frequencies of the operating modes are tested to be 5768.407 Hz and 5771.116Hz and the resonant wavelength change Δ*X* was 0.08 nm for 45° rotation angle. The resonant wavelength changes from −0.071 nm to 0.080 μm, which has a good linear response in working range. The MOEMS-RG with optimized disc sensitive structure can detect the deformation of sensitive membrane effectively, and has a high sensitivity. This resonator shows very large meff, low $f_{0}$, and very high Q. Therefore, it can provide small ${ARW}_{B}$($0.09{}^{\circ}/\sqrt{h}$), which makes it a promising candidate for batch-fabricated, low cost, small size inertial-grade MOEMS gyroscope.

## Figures and Tables

**Table 1:** Frequency split and relative parameters of the MOEMS-RG.

*ω*/Hz	*ψ*_1_/deg	*σ*_c_/Hz	*σ*_s_/Hz
11.7328	7.6335	10.9182	5.9218

**Table 2:** Key design parameters of disc sensitive structure of MOEMS.

Parameter	Value	Parameter	Value
*D*	450 (μm)	*st*	5 (μm)
*d*	150 (μm)	*rt*	5 (μm)
*d*’	15 (μm)	*sw*	5 (μm)
*h*	40 (μm)	Number of rings *N*	15
*y*	207.5 (μm)	Number of spokes *N_s_*	16

**Table 3:** Key character parameters of the MOEMS-RG.

Character ^1^	MOEMS-RG
$f_{0}$	13,756.005 Hz
Q(2Pa)	71,500
*τ*(2Pa)	1.705s
$m_{\mathrm{eff}}$	0.47mg
γ	0.37 mg
${ARW}_{B}$ ^2^	$0.09{}^{\circ}/\sqrt{h}$

^1^ All the parameters are based on the n = 2 mode. ^2^ Calculated based on the assumption that the driving amplitude is 2.5 μm.
