# Correction: Shen, X. et al. Research on the Disc Sensitive Structure of a Micro Optoelectromechanical System (MOEMS) Resonator Gyroscope. *Micromachines*, 2019, *10*, 264

**DOI:** 10.3390/mi10050328

**Published:** 2019-05-16

**Authors:** Xiang Shen, Liye Zhao, Dunzhu Xia

**Affiliations:** Key Laboratory of Micro-Inertial Instrument and Advanced Navigation Technology, Ministry of Education, School of Instrument Science and Engineering, Southeast University, Nanjing 210096, China; 230179630@seu.edu.cn (X.S.); xiadz_1999@163.com (D.X.)

In the published paper [1], Figure 15 should be corrected as follows:

The original sentences for Figure 15 were described as follows:

“The epi-seal process starts with a 40 μm-thick (100) SOI (silicon-on-insulator) wafer with a 2 μm-thick buried oxide layer. The devices were patterned and etched using DRIE (deep reactive ion etching). Then, the trenches were filled with a 2 μm-thick sacrificial LPCVD (low-pressure chemical vapor deposition) oxide layer, and contact holes were subsequently etched into this oxide. These contact holes provide electrical access to the device structure and electrodes, creating a mechanical anchor. A first encapsulation layer (6 μm) was epitaxially grown on top of the sacrificial oxide, and vent holes were etched. Vapor-phase hydrofluoric (HF) was then used to etch the oxide and release the device structure. A thick (20 μm) second encapsulation layer was deposited epitaxially to seal the vent holes and create the hermetic cavity. An aluminum electrical contact was patterned and deposited. Final annealing was performed in a low-temperature (400 °C) nitrogen environment to diffuse the residual hydrogen gas out from the cavity, providing a low-pressure, oxide-free environment that is less than 10 Pa, which yields high Q devices.”

These sentences should be corrected as follows:

“The micro optoelectromechanical system resonator gyroscope (MOEMS-RG) of multiple rings can be fabricated by a conventional three-mask silicon-on-glass (SOG) process. Firstly, a plasma-enhanced chemical vapor deposition (PECVD) SiO_2_ layer is deposited on the back side of the silicon wafer and patterned by a mask to define the anchor. Then, the central post is formed by deep reactive ion etching (DRIE), and the gap between the glass substrate and device is created at the same time. Next, the photoresist is patterned by mask, and then the Cr/Au is deposited in the pattern to form the electrical interconnections and pads through the lift-off process. After that, the Si-glass anodic bonding is performed, and then the silicon wafer is thinned with the chemical mechanical polishing (CMP) process. Finally, the PECVD SiO_2_ layer is deposited on the front side of the silicon wafer and patterned by a mask, and the resonator structure and electrodes are simultaneously released using the Bosch ICP process.”

The changes do not affect the scientific results. We apologize for any inconvenience caused to the readers by these errors. The manuscript will be updated, and the original will remain online on the webpages for the article including a reference to this Correction.

## Figures and Tables

**Figure 15:**
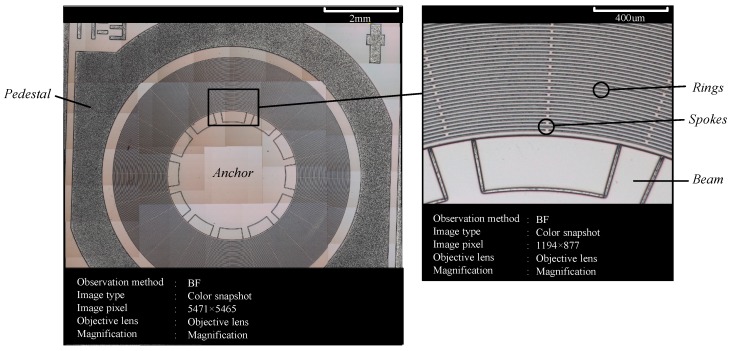
The photo of the fabricated micro optoelectromechanical system resonator gyroscope (MOEMS-RG).
